# Value Cocreation and Codestruction in Digital Health Services: Protocol for a Systematic Review

**DOI:** 10.2196/63015

**Published:** 2025-01-14

**Authors:** Elina Laukka, Tuure Tuunanen, Miia Jansson, Minna Vanhanen, Nina Hirvonen, Jenni Palukka, Märt Vesinurm, Paulus Torkki

**Affiliations:** 1 Department of Public Health Faculty of Medicine University of Helsinki Helsinki Finland; 2 School of Culture and Wellbeing Oulu University of Applied Sciences Oulu Finland; 3 Faculty of Information Technology University of Jyväskylä Jyväskylä Finland; 4 Department of Informatics and Media Uppsala University Uppsala Sweden; 5 Research Unit of Health Sciences and Technology University of Oulu Oulu Finland; 6 RMIT University Melbourne Australia; 7 Focus Area for Digital Solutions Centre for Research and Innovation Oulu University of Applied Sciences Oulu Finland; 8 Institute of Healthcare Engineering and Management at the Department of Industrial Engineering and Management Aalto University School of Science Espoo Finland

**Keywords:** value cocreation, value codestruction, telemedicine, eHealth, systematic review

## Abstract

**Background:**

To successfully design, develop, implement, and deliver digital health services that provide value, they should be cocreated with patients. However, occasionally, the value may also be codestructed. In the field of health care, the concepts of value cocreation and codestruction still need to be better established within emerging digital health services. Studying these concepts is essential for developing effective and sustainable patient-centered care.

**Objective:**

The aim of the study is (1) to understand the antecedents, decisions, and outcomes of value cocreation and codestruction in digital health services, (2) to define the dynamics between value cocreation and codestruction, and (3) to map future research areas of value cocreation and codestruction within digital health services.

**Methods:**

The systematic review will be conducted in accordance with the Joanna Briggs Institute methodology for mixed method systematic reviews and the PRISMA (Preferred Reporting Items for Systematic Reviews and Meta-Analyses) statement. The review considers scientific qualitative, quantitative, or mixed method studies published in English, Finnish, or Swedish that concern either value cocreation or codestruction in digital health services. Studies focusing on physical robotics and online health communities, as well as non–peer-reviewed and nonscientific papers, will be excluded. The searches were conducted using Scopus and MEDLINE during this protocol creation. Critical appraisal will be done using suitable checklists for qualitative, quantitative, and mixed method studies. The review will adhere to a convergent integrated approach as outlined in the Joanna Briggs Institute methodology for mixed methods systematic reviews.

**Results:**

The searches resulted in a total of 837 records. The antecedents, decisions, and outcomes of value cocreation and codestruction in the context of digital health services will be described in a finalized systematic review. In the outcomes, our main interest is the effect on patient outcomes and experiences and professional experiences.

**Conclusions:**

Since our study involves diverse scientific fields, there is a risk that our search does not capture all relevant papers. To mitigate this risk, we used 2 large databases for the searches. In addition, the value cocreation or codestruction terms may not have been used in all studies focusing on the collaborative roles of patients and providers, especially in the medical field, and that may be difficult to capture. The review reveals the current understanding of value cocreation and codestruction in digital health services and shapes the research agenda for these phenomena. Value cocreation can be used to both design and efficiently use digital health services trying to maximize the value for patients.

**International Registered Report Identifier (IRRID):**

DERR1-10.2196/63015

## Introduction

Value cocreation and customers’ interactive roles in the service exchange have been essential to understanding how value is created in services [1]. Rapid technological advances and digital transformation are transforming the context in which value is created [2]. Health care services had a significant transformation as digital health services rapidly expanded during the COVID-19 pandemic [3,4]. Digital health services can be defined as the use of information and communication technologies in health care products, services, and processes [5]. Digital services, including telemedicine interventions, mobile health apps, and remote monitoring devices, have been suggested as a potential solution to tackle issues related to accessibility, availability, and health care costs [6,7]. In addition to the technical development, it also changes the way of thinking and how services are provided and perceived [4].

Cocreation, a burgeoning paradigm within management literature, facilitates the joint creation of value by enterprises and consumers through interactive processes [8]. Since the onset of the 21st century, the concept of cocreation has proliferated extensively, evidenced in scholarly discourse and empirical inquiries, thereby challenging established tenets of capitalist economies. In such economies, value traditionally tends to be predetermined prior to market transactions [8,9]. Health care is a complex service where defining and operationalizing value presents significant challenges [10,11]. Since Porter [12] introduced the idea of measuring health care value as patient-relevant outcomes per costs associated with the health problem, the concept has been widely discussed for its potential to unify the objectives of various stakeholders [11]. In the context of digital health services, value may be more closely related to outcomes and experiences, with costs being more pertinent at the system level.

From the standpoint of cocreation, suppliers and customers are no longer perceived as adversaries but rather as collaborators engaging with each other to foster the emergence of novel business prospects [13]. During the last decade, Yi and Gong [14] established the significance of value cocreation behavior, which provides a multidimensional framework encompassing diverse value cocreation activities, such as information seeking, information sharing, responsible behavior, and personal interaction, that help explain how customers and firms, or patients and health care providers in this case, interact and collaborate.

Additionally, the digitalization of services within health care ecosystems is altering how value is created, delivered, experienced, and evaluated [15,16]. Understanding value cocreation and the interactive roles of customers in the service exchange is crucial for comprehending how value is created in services [1], with value cocreation being conceptualized as a joint problem-solving process [17]. For instance, value cocreation in digital health services can be depicted through customer engagement, encompassing emotional, active, and cognitive dimensions [18].

However, value is not always cocreated. Value codestruction signifies that not all interactions and relationships yield positive or value-creating results; occasionally, these engagements may even lead to adverse outcomes [19,20]. Value codestruction refers to an interactive process between service systems that leads to a reduction in the well-being of at least 1 of the systems, which can affect either individuals or organizations depending on the nature of the service system [20]. For example, value codestruction may manifest in the inability to search for, understand, and use health information gathered on the web [21]. Additionally, the assumption that telemedicine can negatively impact doctor-patient relationships inevitably leads to value-in-use destruction [21].

As digital services differ significantly from traditional services, there is a need to understand how to enhance value cocreation between a service provider and its users in digital services [22,23]. Tuunanen et al [23] identified 5 mechanisms to support value cocreation in the design of digital services, namely social use, customer orientation and decision-making, service experience, service use context, and customer values and goals.

In response to evolving population needs, it is evident that the role of patients within digital health services and health care at large has transitioned toward a collaborative partnership between professionals and patients [24,25]. According to Huber et al [26], the definition of health is also changing from “complete mental, physical, and social well-being” toward “the ability to self-manage and adapt.” According to van Druten et al [27], a similar multifaceted approach to Huber’s concept of positive health was shared by many perspectives. Nevertheless, upon closer examination, it was observed that the core elements of positive health, namely “the ability to adapt and to self-manage,” were also acknowledged in other health concepts, regardless of perspective. These health concepts described “the ability to adapt” as, for instance, adjusting to changing physical conditions like aging, illness, or disability, maintaining emotional balance, and viewing health as a dynamic state requiring adaptation to circumstances. “The ability to self-manage” was often described as autonomy or independence. Through the value cocreation process, professionals and patients can make a significant contribution to health outcomes as partners [28]. For example, patients can provide perspectives on areas of the care process that are invisible to health care professionals [29,30]. Previous literature has discussed many consequences of value cocreation related to health outcomes, service experience, perceived service quality, and service engagement [31].

While value cocreation has also been studied in health care, less attention has been given to the investigation of value codestruction [29,32]. As the field of health care is constantly becoming more digitalized, examining value cocreation and value codestruction in digital services would provide valuable insights into developing such services. Peng et al [31] conducted a systematic review of value cocreation in health care and digital services before the COVID-19 pandemic. They also encouraged researchers to explore further opportunities for value cocreation in both web-based and hybrid environments. Even so, the review by Peng et al [31] did not address value codestruction, which, in conjunction with value cocreation, could aid in enhancing the efficiency of digital health services. Several authors have highlighted the necessity of gaining a more comprehensive understanding of value codestruction and integrating it with the research on value cocreation [33,34]. Considering the pandemic’s impact on digital service expansion [3,4], increasing resource constraints due to rising chronic illnesses, and aging populations [35,36], conducting a new systematic literature review could provide a contemporary perspective on value cocreation in health care. Furthermore, this review aims to provide a more comprehensive understanding of value codestruction.

So as to gain a more current understanding of both value cocreation and value codestruction in digital health services, this review seeks to identify scientific studies published between 2020 and 2024, using the Joanna Briggs Institute’s (JBI) guidance for mixed method systematic reviews [37] and adhering to the PRISMA (Preferred Reporting Items for Systematic Reviews and Meta-Analyses) checklist for reporting systematic reviews [38] The objectives of the review will be (1) to understand the antecedents, decisions, and outcomes (ADO) of value cocreation and value codestruction, (2) to define the dynamics between value cocreation and value codestruction in digital health services, and (3) to map future research areas of value cocreation and value codestruction within digital health services. To answer the first research question, this review uses a systematic literature review framework, namely, the ADO framework by Paul and Benito [39]. The ADO framework aims to identify the known aspect of any phenomenon, which in the case of this review is value cocreation and value codestruction in the context of digital health services. Our research questions are (1) What are the ADO of value cocreation and value codestruction in digital health services? (2) What are the dynamics between value cocreation and value codestruction in digital health services? (3) What are the most promising future research areas in value cocreation and value codestruction within digital health services?

## Methods

### Overview

The systematic review will be conducted in accordance with the JBI methodology for mixed method systematic review [37] and the PRISMA statement [38,40] (Multimedia Appendix 1). This protocol has been registered in PROSPERO (International Prospective Register of Systematic Reviews; 549303). During this review protocol, the searches have been conducted, but the screening, quality assessment, and analysis will be carried out in the finalized systematic review.

### Search Strategy

Using the search strategy developed by Peng et al [31] as a foundation, adjustments were made to include value codestruction. The search strategy aimed to identify peer-reviewed scientific studies and was conducted in 3 steps. First, to ensure an optimal search strategy for both value cocreation and value codestruction, an initial limited search of MEDLINE and Scopus was conducted on May 21, 2024, which resulted in 48 and 247 papers, respectively. MEDLINE and Scopus were selected since they collectively provide extensive coverage of publications on digital health services, value cocreation, and value codestruction. Second, relevant papers were identified through title, abstract, and index term analysis. An information specialist was consulted during the development of the final search strategy. Keywords were truncated as necessary, and index terms such as MeSH were used in MEDLINE (Multimedia Appendix 2). Additionally, the reference lists of all included studies will be screened to identify additional relevant studies.

### Eligibility Criteria

This study will include studies that investigate either value cocreation, value codestruction, or both in digital health services. This review will consider scientific qualitative, quantitative, or mixed method studies. Papers published in English, Finnish, and Swedish will be eligible for inclusion. Only papers published between January 1, 2020, and the present (June 2024) concerning value cocreation were considered, as an earlier review covered the period from 2008 to 2019 [31]. Regarding value codestruction, the limitation period extends from January 1, 2008, to December 31, 2008, since the earlier review did not address value codestruction. Studies focusing on physical robotics, as well as non–peer-reviewed and nonscientific papers, will be excluded.

### Study Selection

The results of the search are presented in a PRISMA flow diagram [38]. All citations identified through the described search strategy were compiled and uploaded into Rayyan (Al Rayyan Company), which was also used to remove duplicate entries. Titles and abstracts will then undergo independent screening by 2 team members (NH and JP) using predefined inclusion and exclusion criteria. For papers lacking abstracts, the full text will be obtained. Following the title and abstract screening, potentially relevant studies will be retrieved in full. Two independent reviewers (NH and JP) will thoroughly assess these studies and determine their suitability based on the inclusion criteria. Exclusion reasons will be documented for studies that do not meet the inclusion criteria. Any discrepancies during the study selection or any other process will be resolved through discussion or by consulting a third team member (EL and PT). All search methods, strategies, and sources will be detailed or named in the final report, ensuring replicability.

### Assessment of Methodological Quality

Before being included in the review, papers will undergo evaluation by 2 separate reviewers (EL and M Vanhanen) to ensure methodological soundness. We will use the JBI checklists for qualitative and quantitative studies and the Mixed Methods Appraisal Tool for mixed method studies [41]. In cases where necessary, authors of papers will be contacted to request missing or additional data to ensure clarity. The outcomes of critical appraisal will be presented both in narrative form and in a table format. Data extraction and synthesis will be conducted for all studies, irrespective of their methodological quality assessment outcomes, whenever feasible.

### Data Extraction and Synthesis

Two independent reviewers (EL and M Vanhanen) will extract both quantitative and qualitative data from the studies included in the review. The extracted data will encompass specific details about populations, study methods, phenomena of interest, context, and outcomes relevant to the review questions. Quantitative data will include outcomes derived from descriptive and inferential statistical tests. Additionally, qualitative data will comprise verbatim themes or subthemes accompanied by corresponding illustrations and will be assigned a level of credibility. Authors of papers will be contacted up to a maximum of 2 times to request missing or additional data, as necessary.

### Data Transformation

The quantitative data will undergo a process of “qualitization,” which entails transforming it into textual descriptions or narrative interpretations that directly address the review questions.

### Data Synthesis and Integration

This review will adhere to a convergent integrated approach as outlined in the JBI methodology for mixed methods systematic reviews. This approach involves integrating the qualitized data with the qualitative data. The assembled data will be categorized and pooled based on similarity in meaning, ultimately generating a set of integrated findings presented as the line of action statements. We will classify the data using the ADO framework [39]. Additionally, we will synthesize the current knowledge of each dimension and formulate a future research agenda based on the findings.

### Ethical Considerations

Since concept analyses solely rely on secondary publicly available data sourced from primary research studies, there is no requirement for research ethics approval.

### Validity and Rigor

The following activities will be performed to enhance the review’s validity and rigor:

1. Method: The systematic review will be conducted following the JBI guidelines [37] and following the PRISMA statement [38].
2. Search: To increase the reliability of the review, an information specialist with expertise in health care, value cocreation, and value codestruction will be consulted. Additionally, several database sources will be included in the final search to ensure the richness of the data to be analyzed.
3. Screening, data extraction, and synthesis: Each of the previously mentioned phases will be conducted independently by at least 2 independent team members (NH and JP), which will enhance the reliability of the review.
4. Process: The research team members (EL, TT, M Vanhanen, NH, JP, MJ, M Vesinurm, and PT) will continuously review the paper during ongoing meetings throughout the process.

## Results

**Figure 1:**
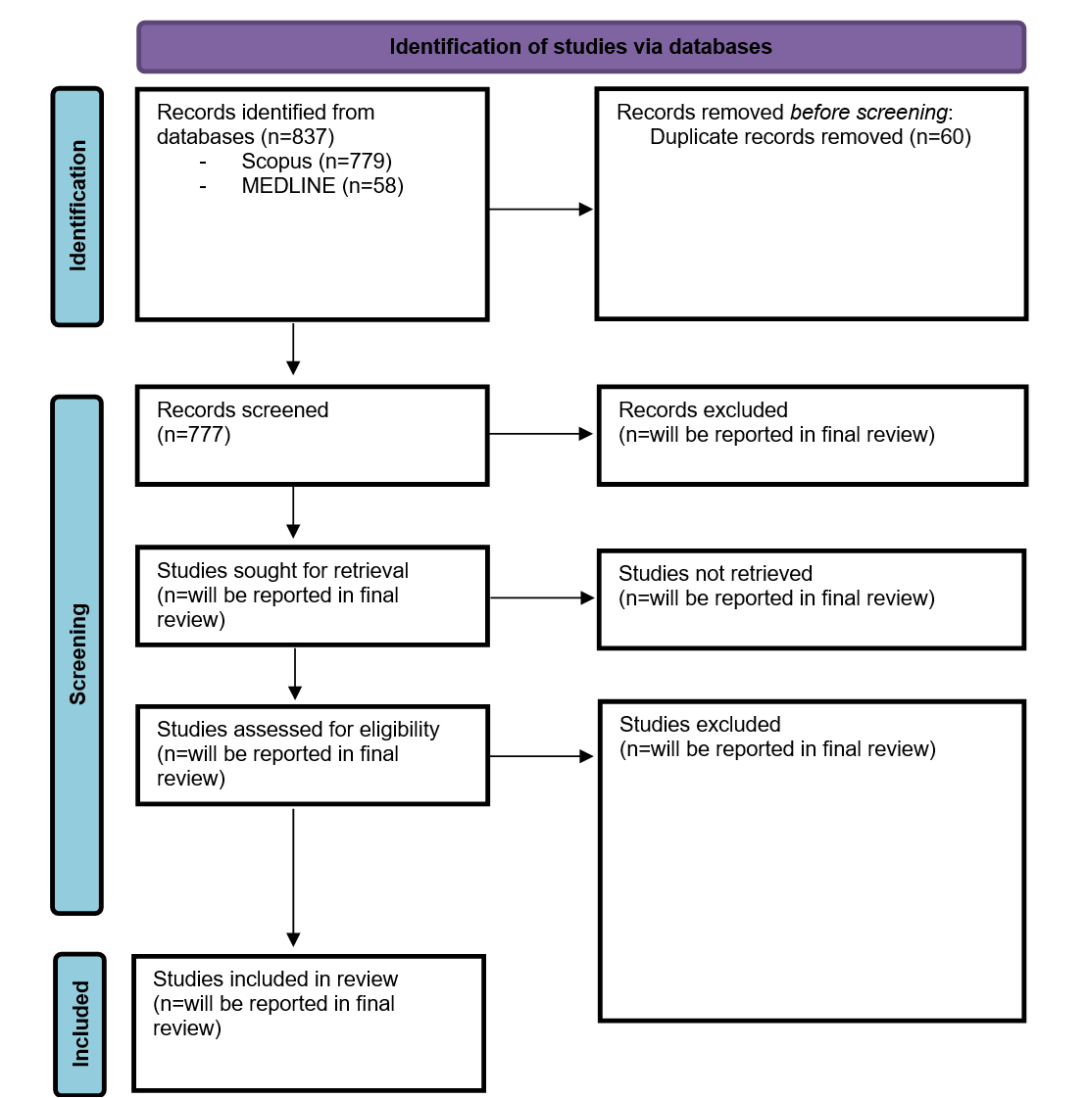
PRISMA (Preferred Reporting Items for Systematic Reviews and Meta-Analyses) flow diagram.

The review started in May 2024 and will be completed in a time frame of 8 months. This time phase includes the following phases: screening, data extraction, quality assessment, and data synthesis. The literature search was conducted entirely during the review protocol process and reported in the PRISMA diagram. The final search in MEDLINE and Scopus on June 6, 2024, resulted in 58 and 770 papers, respectively (Figure 1). As Peng et al [31] omitted value codestruction in their search, a supplementary search focusing exclusively on value codestruction was conducted on September 19, 2024, covering the years 2008 to 2019. This search yielded 9 papers from Scopus and none from MEDLINE. In total, 837 records were detected. The systematic review is anticipated to be ready for submission by December 2024.

## Discussion

### Principal Findings

Our review will reveal the current understanding of value cocreation and codestruction in digital health services and shape the research agenda for these phenomena. In their review, Peng et al [31] focused on studies published prior to the COVID-19 pandemic, a period after which the number of digital health services has significantly increased. Consequently, our review will provide the most recent insights into value cocreation and value codestruction within digital health services. The ADO framework [39] assists in producing a knowledge map outlining the associations between ADO of value cocreation and codestruction in the context of digital health services. The ADO of value cocreation and codestruction will be described. In the outcomes, our main interest is the effect on patient outcomes and experiences and professional experiences. The more detailed results will be determined based on the finalized review.

Value cocreation can be used in both designing as well as the efficient use of digital health services trying to maximize value for patients. In other digital services, value cocreation and codestruction have been studied more thoroughly, as most of the services nowadays are digital, and the world has already been described as digital-first [42].

As the field of health care is constantly more digitalized, examining value cocreation and codestruction in digital services would provide valuable insights into developing such services. To support the cost-effectiveness of the services and try to minimize the effects of digital exclusivity, it is crucial to understand value cocreation and codestruction better in digital health services.

### Strengths and Limitations

Since our study is crossing the scientific fields, there is a risk that our search does not capture all relevant papers. To mitigate this risk, we include multiple databases for the searches. However, only peer-reviewed studies will be included in this review, which excludes gray literature [43]. In addition, the value cocreation or codestruction terms may not have been used in all studies focusing on the collaborative roles of patients and providers, especially in the medical field, and that may be difficult to capture.

## Appendix

Multimedia Appendix 1 PRISMA-S (Preferred Reporting Items for Systematic reviews and Meta-Analyses Literature Search Extension) checklist.

Multimedia Appendix 2 Search strategy.

## Abbreviations

ADO: antecedents, decisions, and outcomes
JBI: Joanna Briggs Institute
PRISMA: Preferred Reporting Items for Systematic Reviews and Meta-Analyses
PROSPERO: International Prospective Register of Systematic Reviews

## Data Availability

The datasets generated and analyzed during this study are available from the corresponding author on reasonable request.

## References

[1] Lumivalo J, Tuunanen T, Salo M (2023). Value co-destruction: a conceptual review and future research agenda. J Serv Res.

[2] Jain S, Sharma K, Devi S (2023). The dynamics of value co‐creation behavior: a systematic review and future research agenda. Int J Consum Stud.

[3] Ndayishimiye C, Lopes H, Middleton J (2023). A systematic scoping review of digital health technologies during COVID-19: a new normal in primary health care delivery. Health Technol (Berl).

[4] Rosenlund M, Kinnunen UM, Saranto K (2023). The use of digital health services among patients and citizens living at home: scoping review. J Med Internet Res.

[5] Chidambaram S, Jain B, Jain U, Mwavu R, Baru R, Thomas B, Greaves F, Jayakumar S, Jain P, Rojo M, Battaglino MR, Meara JG, Sounderajah V, Celi LA, Darzi A (2024). An introduction to digital determinants of health. PLOS Digit Health.

[6] Golinelli D, Boetto E, Carullo G, Nuzzolese AG, Landini MP, Fantini MP (2020). Adoption of digital technologies in health care during the COVID-19 pandemic: systematic review of early scientific literature. J Med Internet Res.

[7] Petracca F, Ciani O, Cucciniello M, Tarricone R (2020). Harnessing digital health technologies during and after the COVID-19 pandemic: context matters. J Med Internet Res.

[8] Prahalad CK, Ramaswamy V (2004). Co-creation experiences: the next practice in value creation. J Interact Mark.

[9] Vargo SL, Lusch RF (2004). Evolving to a new dominant logic for marketing. J Mark.

[10] Sánchez-Fernández R, Iniesta-Bonillo MÁ, Holbrook MB (2018). The conceptualisation and measurement of consumer value in services. Int J Mark Res.

[11] Landon SN, Padikkala J, Horwitz LI (2021). Defining value in health care: a scoping review of the literature. Int J Qual Health Care.

[12] Porter ME (2010). What is value in health care?. N Engl J Med.

[13] Galvagno M, Dalli D (2014). Theory of value co-creation: a systematic literature review. Manag Serv Qual.

[14] Yi Y, Gong T (2013). Customer value co-creation behavior: scale development and validation. J Bus Res.

[15] Pal S, Biswas B, Gupta R, Kumar A, Gupta S (2023). Exploring the factors that affect user experience in mobile-health applications: a text-mining and machine-learning approach. J Bus Res.

[16] Kraus S, Schiavone F, Pluzhnikova A, Invernizzi AC (2021). Digital transformation in healthcare: analyzing the current state-of-research. J Bus Res.

[17] Aarikka-Stenroos L, Jaakkola E (2012). Value co-creation in knowledge intensive business services: a dyadic perspective on the joint problem solving process. Ind Mark Manag.

[18] Lo Presti L, Testa M, Marino V, Singer P (2019). Engagement in healthcare systems: adopting digital tools for a sustainable approach. Sustainability.

[19] Echeverri P, Skålén P (2011). Co-creation and co-destruction: a practice-theory based study of interactive value formation. Mark Theory.

[20] Plé L, Chumpitaz Cáceres R (2010). Not always co‐creation: introducing interactional co‐destruction of value in service‐dominant logic. J Serv Mark.

[21] Marsilio M, Mastrodascio M (2024). Technology-enabled value co-creation in healthcare: a configurational approach. Public Manag Rev.

[22] Čaić M, Odekerken-Schröder G, Mahr D (2018). Service robots: value co-creation and co-destruction in elderly care networks. J Serv Manag.

[23] Tuunanen T, Lumivalo J, Vartiainen T, Zhang Y, Myers MD (2023). Micro-level mechanisms to support value co-creation for design of digital services. J Serv Res.

[24] Richards T, Montori VM, Godlee F, Lapsley P, Paul D (2013). Let the patient revolution begin. BMJ.

[25] Popa V, Geissler J, Vermeulen R, Priest E, Capperella K, Susuzlu G, Terry SF, Brooke N (2024). Delivering digital health solutions that patients need: a call to action. Ther Innov Regul Sci.

[26] Huber M, Knottnerus JA, Green L, van der Horst H, Jadad AR, Kromhout D, Leonard B, Lorig K, Loureiro MI, van der Meer JWM, Schnabel P, Smith R, van Weel C, Smid H (2011). How should we define health?. BMJ.

[27] van Druten VP, Bartels EA, van de Mheen D, de Vries E, Kerckhoffs APM, Nahar-van Venrooij LMW (2022). Concepts of health in different contexts: a scoping review. BMC Health Serv Res.

[28] von Thiele Schwarz U (2016). Co-care: producing better health outcome through interactions between patients, care providers and information and communication technology. Health Serv Manage Res.

[29] Keeling DI, Keeling K, de Ruyter K, Laing A (2020). How value co-creation and co-destruction unfolds: a longitudinal perspective on dialogic engagement in health services interactions. J Acad Mark Sci.

[30] Elg M, Engström J, Witell L, Poksinska B (2012). Co-creation and learning in health-care service development. J Serv Manag.

[31] Peng Y, Wu T, Chen Z, Deng Z (2022). Value cocreation in health care: systematic review. J Med Internet Res.

[32] Kaartemo V, Känsäkoski H (2018). Information and knowledge processes in health care value co-creation and co-destruction. Sage Open.

[33] Peng J, Yang X, Poon P, Xie L (2022). Enhancing users' well-being in virtual medical tourism communities: a configurational analysis of users’ interaction characteristics and social support. Technol Soc.

[34] Zyzak B, Martinussen PE (2024). OK computer: applying the public service logic on digital health services. Public Manag Rev.

[35] Murthy VNR, Okunade AA (2016). Determinants of U.S. health expenditure: evidence from autoregressive distributed lag (ARDL) approach to cointegration. Econ Model.

[36] Keehan SP, Cuckler GA, Sisko AM, Madison AJ, Smith SD, Stone DA, Poisal JA, Wolfe CJ, Lizonitz JM (2015). National health expenditure projections, 2014-24: spending growth faster than recent trends. Health Aff (Millwood).

[37] Lizarondo L, Stern C, Carrier J, Godfrey C, Rieger K, Salmond S, Apostolo J, Kirkpatrick P, Loveday H, Aromataris E, Lockwood C, Porritt C, Pilla B, Jordan Z (2020). Chapter 8: Mixed methods systematic reviews. JBI Manual for Evidence Synthesis.

[38] Page MJ, McKenzie JE, Bossuyt PM, Boutron I, Hoffmann TC, Mulrow CD, Shamseer L, Tetzlaff JM, Akl EA, Brennan SE, Chou R, Glanville J, Grimshaw JM, Hróbjartsson A, Lalu MM, Li T, Loder EW, Mayo-Wilson E, McDonald S, McGuinness LA, Stewart LA, Thomas J, Tricco AC, Welch VA, Whiting P, Moher D (2021). The PRISMA 2020 statement: an updated guideline for reporting systematic reviews. BMJ.

[39] Paul J, Benito GRG (2017). A review of research on outward foreign direct investment from emerging countries, including China: what do we know, how do we know and where should we be heading?. Asia Pac Bus Rev.

[40] Rethlefsen ML, Kirtley S, Waffenschmidt S, Ayala AP, Moher D, Page MJ, Koffel JB (2021). PRISMA-S: an extension to the PRISMA statement for reporting literature searches in systematic reviews. Syst Rev.

[41] Hong QN, Fàbregues S, Bartlett G, Boardman F, Cargo M, Dagenais P, Gagnon MP, Griffiths F, Nicolau B, O’Cathain A, Rousseau MC, Vedel I, Pluye P (2018). The Mixed Methods Appraisal Tool (MMAT) version 2018 for information professionals and researchers. Educ Inf.

[42] Baskerville RL, Myers MD, Yoo Y (2020). Digital first: the ontological reversal and new challenges for information systems research. MIS Q.

[43] Aromataris E, Lockwood C, Porritt K, Pilla B, Jordan Z (2024). JBI Manual for Evidence Synthesis.

