# Supplementary material for: Value Cocreation and Codestruction in Digital Health Services: Protocol for a Systematic Review
Source: JMIR Res Protoc. 2025 Jan 14;14:e63015. doi: 10.2196/63015 (PMC11775491; doi:10.2196/63015)
Supplement: Multimedia Appendix 1 [file resprot_v14i1e63015_app1.doc]

**PRISMA-S: an extension to the PRISMA Statement for Reporting Literature Searches in Systematic Reviews**

| Section/topic | # | Checklist item | Location(s) Reported |
| --- | --- | --- | --- |
| **INFORMATION SOURCES AND METHODS** | | | |
| Database name | 1 | Name each individual database searched, stating the platform for each | Databases (Scopus and MEDLINE) mentioned in Methods -section under Search strategy |
| Multi-database searching | 2 | If databases were searched simultaneously on a single platform, state the name of the platform listing all the database searched | No multi-databases were used. |
| Study registries | 3 | List any registries searches. | No study registries were used as the studies needed to be peer-reviewed scientific journals. |
| Online sources and browsing | 4 | Describe any online or print source purposefully searched or browsed (e.g., tables of contents, print conference proceedings, websites), and show how this was done. | No online sources or browsing were used as the studies needed to be peer-reviewed scientific journals. |
| Citation searching | 5 | Indicate whether cited references or citing references were examined, and describe any methods used for locating cited/citing references (e.g., browsing reference lists, using a citation index, setting up email alerts for references citing included studies). | This phase has been mentioned in Methods under Search strategy. This phase will be reported in finalized systematic review. |
| Contacts | 6 | Indicate whether additional studies or data were sought by contacting authors, experts, manufacturers, or others. | No applicable at this point of the review. This will be reported in finalized systematic review. |
| Other methods | 7 | Describe any additional information sources or search methods used. | No additional information sources or search methods were or will be used. |
| **SEARCH STRATEGIES** | | | |
| Full search strategy | 8 | Include the search strategies for each database and information source, copied and pasted exactly as run. | Full search strategy available in Multimedia Appendix 2. |
| Limits and restrictions | 9 | Specify that no limits were used, or describe any limits or restrictions applied. | Limitations described in Appendix 2 and in Methods under Eligibility criteria. |
| Search filters | 10 | Indicate whether published search filters were used (as originally designed or modified), and if so, cite the filter(s) used. | Limitations described in Appendix 2 and in Methods under Eligibility criteria. |
| Prio work | 11 | Indicate when search strategies from other literature reviews were adopted or reused for a substantive part or all of the search, citing the previous review(s). | This review utilizes search strategy used by Peng et al. [31] and this has been reported in Methods under Search strategy. |
| Updates | 12 | Report the methods used to update the search(es) (e.g., rerunning searches, email alerts). | Searches have not been updated. |
| Dates of searched | 13 | For each search strategy, provide the date when last search occurred. | The dates are provided. |
| Rethlefsen ML, Kirtley S, Waffenschmidt S, Ayala AP, Moher D, Page MJ, Koffel JB; PRISMA-S Group. PRISMA-S: an extension to the PRISMA Statement for Reporting Literature Searches in Systematic Reviews. Syst Rev. 2021;10(1):39. doi: [10.1186/s13643-020-01542-z](https://doi.org/10.1186/s13643-020-01542-z) | | | |
