# Supplementary material for: Value Cocreation and Codestruction in Digital Health Services: Protocol for a Systematic Review
Source: JMIR Res Protoc. 2025 Jan 14;14:e63015. doi: 10.2196/63015 (PMC11775491; doi:10.2196/63015)
Supplement: Multimedia Appendix 2 [file resprot_v14i1e63015_app2.doc]

**Search strategy of Scopus**

| **Search (Scopus)** | **Query** | **Filters** | **Records retrieved** |
| --- | --- | --- | --- |
| #1 | TITLE-ABS-KEY ( patient* OR health OR medical OR "health care" OR "healthcare" OR "online health community*" OR ehealth OR e-health OR mhealth OR m-health OR "mobile health" OR telemedicine OR telehealth OR ( smart AND *care ) OR digital* OR ( remote AND *care ) ) | Article title  Abstract  Keywords | 18,653,544 |
| #2 | TITLE-ABS-KEY ( ( "value co-creat*" OR "value cocreat*" OR "value-co-creat*" OR "co-creat* of value" OR "co-creat* value" ) ) | Article title  Abstract  Keywords | 5,181 |
| #3 | TITLE-ABS-KEY ( ( "value co-destr*" OR "value codestr*" OR "value-co-destr*" OR "co-destr* of value" OR "co-destr* value" ) ) | Article title  Abstract  Keywords | 202 |
| #4 | #1 AND (#2 OR #3) | | 1,216 |
| Limited to 1/2020-6/2024 | | | 786 |
| Languages: English, Finnish, Swedish | | | 770 |

| **Search (Scopus)** | **Query** | **Filters** | **Records retrieved** |
| --- | --- | --- | --- |
| #1 | TITLE-ABS-KEY ( patient* OR health OR medical OR "health care" OR "healthcare" OR "online health community*" OR ehealth OR e-health OR mhealth OR m-health OR "mobile health" OR telemedicine OR telehealth OR ( smart AND *care ) OR digital* OR ( remote AND *care ) ) | Article title  Abstract  Keywords | 18,653,544 |
| #2 | TITLE-ABS-KEY ( ( "value co-destr*" OR "value codestr*" OR "value-co-destr*" OR "co-destr* of value" OR "co-destr* value" ) ) | Article title  Abstract  Keywords | 202 |
| #3 | #1 AND #2 and Limited to 1/2008-12/2019 | | 9 |
| Languages: English, Finnish, Swedish | | | 9 |

**Search strategy of MEDLINE**

| **Search (MEDLINE)** | **Query** | **Filters** | **Records retrieved** |
| --- | --- | --- | --- |
| #1 | ( patient*[Title/Abstract] OR health[Title/Abstract] OR medical[Title/Abstract] OR "health care"[Title/Abstract] OR "healthcare"[Title/Abstract] OR "online health community*"[Title/Abstract] OR ehealth[Title/Abstract] OR e-health[Title/Abstract] OR mhealth[Title/Abstract] OR m-health[Title/Abstract] OR "mobile health" [Title/Abstract] OR telemedicine [Title/Abstract] OR telehealth [Title/Abstract] OR "smart *care" [Title/Abstract] OR digital* [Title/Abstract] OR "remote *care"[Title/Abstract]) | Article title  Abstract  Keywords | 11,238,923 |
| #2 | ( "value co-creat*"[Title/Abstract] OR "value cocreat*"[Title/Abstract] OR "value-co-creat*"[Title/Abstract] OR "co-creat* of value"[Title/Abstract] OR "co-creat* value"[Title/Abstract]) | Article title  Abstract  Keywords | 159 |
| #3 | ( "value co-destr*"[Title/Abstract] OR "value codestr*"[Title/Abstract] OR "value-co-destr*"[Title/Abstract] OR "co-destr* of value"[Title/Abstract] OR "co-destr* value"[Title/Abstract]) | Article title  Abstract  Keywords | 3 |
| #4 | #1 AND (#2 OR #3) | | 81 |
| Limited to date 1/2020-6/2024 | | | 58 |
| Languages: English, Finnish, Swedish | | | 58 |

| **Search (MEDLINE)** | **Query** | **Filters** | **Records retrieved** |
| --- | --- | --- | --- |
| #1 | ( patient*[Title/Abstract] OR health[Title/Abstract] OR medical[Title/Abstract] OR "health care"[Title/Abstract] OR "healthcare"[Title/Abstract] OR "online health community*"[Title/Abstract] OR ehealth[Title/Abstract] OR e-health[Title/Abstract] OR mhealth[Title/Abstract] OR m-health[Title/Abstract] OR "mobile health" [Title/Abstract] OR telemedicine [Title/Abstract] OR telehealth [Title/Abstract] OR "smart *care" [Title/Abstract] OR digital* [Title/Abstract] OR "remote *care"[Title/Abstract]) | Article title  Abstract  Keywords | 11,238,923 |
| #2 | ( "value co-destr*"[Title/Abstract] OR "value codestr*"[Title/Abstract] OR "value-co-destr*"[Title/Abstract] OR "co-destr* of value"[Title/Abstract] OR "co-destr* value"[Title/Abstract]) | Article title  Abstract  Keywords | 3 |
| #3 | #1 AND #2 and Limited to 1/2008-12/2019 | | 0 |
| Languages: English, Finnish, Swedish | | | 0 |
